# Talk COVID to Me: Language Rights and Canadian Government Responses to the Pandemic

**DOI:** 10.1017/S0008423920000359

**Published:** 2020-04-28

**Authors:** Stéphanie Chouinard, Martin Normand

**Affiliations:** 1 Royal Military College (Kingston) and Queen's University, Department of Political Science and Economics, 13, General Crerar Crescent, Kingston (ON), K7K 4B9; 2 School of Political Studies, University of Ottawa, 75 Laurier Avenue East, Ottawa (ON), K1N 6N5

## Abstract

Since the COVID-19 outbreak, a gradual loosening of linguistic obligations in public institutions and governments has been observed in various jurisdictions in Canada. This article argues that in addition to legal requirements to provide minority language services, it is not justifiable for governments to suspend or curtail such services in an emergency situation, for reasons pertaining to public safety and public health. After performing a survey and analysis of government actions against their constitutional, legislative, and policy language obligations to highlight best practices and deficiencies, we discuss the policy implications of these actions. In conclusion, the article considers how governments could better uphold their language obligations in times of emergency.

Since the COVID-19 outbreak, a gradual loosening of linguistic obligations in public institutions and governments has been observed in various jurisdictions in Canada. This article argues that in addition to legal requirements to provide minority language services, it is not justifiable for governments to suspend or curtail such services in an emergency situation, for reasons pertaining to public safety and public health. After performing a survey and analysis of government actions against their constitutional, legislative, and policy language obligations to highlight best practices and deficiencies, we discuss the policy implications of these actions. In conclusion, the article considers how governments could better uphold their language obligations in times of emergency.

## Government Responses to the COVID-19 Pandemic

Canada's language regime draws upon two traditions (Cardinal, 2015). The first is political compromise, which, in practice, has allowed for the recognition of both French and English as a fundamental characteristic of Canadian society. As official languages, they are each a vector for citizenship and political participation. The second is federalism, which, in the spirit of compromise, empowers the federated identities to develop their own sets of language laws, rules, and policies pertaining to the delivery of government services, communications with the public, and supports for Official Languages Minority Communities (OLMCs). In other words, as language is an ancillary jurisdiction in the Canadian federal regime, it has led to a very different status for French, English, and a number of Indigenous languages from one province/territory to the other. Services and communications in non-official languages are offered on the basis of accommodation; they do not stem from a constitutional or legislative obligation. They will therefore be discussed only briefly in this article. The following survey of government responses to the crisis highlights best practices and deficiencies with respect to official language obligations.

### Federal Government

The federal government is bound by the *Official Languages Act* (OLA) to communicate with and serve Canadians in both official languages, and Charter language dispositions would apply if the Emergencies Act were enacted. The federal government has generally provided information in both official languages during the outbreak, for example by using simultaneous interpretation. However, as the situation became more urgent, a gradual easing of language restrictions has occurred. After stocks of sanitizing products were depleted, Health Canada lifted bilingual labeling regulations to allow materials featuring English-only instructions to be distributed (Fortin-Gauthier, 2020). Five weeks later, this directive was extended to apply to all cleaning products. English has been the primary language of the Prime Minister's daily briefings, unequally supplemented by French (Vachet, 2020). He also addressed children of the nation in English only, once on March 22 (Tang, 2020) and then on April 5 (CBC, 2020). Official Languages Minister Mélanie Joly, who is on the emergency response task force, has been mum about these deviations to the norm. Commissioner of Official Languages Raymond Théberge has recently spoken out, reminding the federal government that upholding its language obligations during the outbreak was “a matter of respect and safety for all Canadians,” and announcing that he would publish a special report on the federal government's respect of the OLA in its response to the COVID-19 crisis (OCOL, 2020).

### Ontario

In the realm of language regimes, Ontario is a “pragmatic” province, granting access to French-language services “whenever practical and reasonable” (Cardinal and Normand, 2013: 120). The *French Language Services Act* does not require the provincial government to provide all of its communications in both French and English. However, in 2010, the Treasury Board adopted the Communications in French Directive, following lessons learned from the H1N1 outbreak after French-language documentation had not been distributed. This directive mandates all provincial ministries and agencies to “consider the Francophone community's specific needs” and to “incorporate appropriate approaches into the communications activities, to reach Francophone audiences effectively” (Government of Ontario, 2010). In this respect, the province has not fulfilled its duties. Until the third week of April, daily briefings were held in English only (Gratton, 2020). Written documentation was made available in French hours after publication in English, and there have been delays in posting information in French regarding COVID-19 on the Ontario.ca website. Associate Ombudsman of Ontario Kelly Burke has received complaints about these discrepancies (Carolino, 2020).

### New Brunswick

Of all the language regimes in Canada, New Brunswick's is the most generous to its official-language minority. Canada's only officially bilingual province, New Brunswick adopted legislation in 1981 recognizing the equality of the French- and English-language communities—an act which was entrenched in the constitution in 1993. Perhaps because of these enhanced obligations, linguistic shortcomings in the government response to COVID-19 have been felt more acutely. Despite the availability of simultaneous interpretation during briefings, Premier Blaine Higgs has not made use of the service, and has occasionally refused to answer questions fielded by Francophone reporters (Gravel, 2020). Commissioner of Official Languages Shirley MacLean noted that “the [New Brunswick] *Official Languages Act* applies to institutions, not to elected representatives” (Gravel, 2020; personal translation). However, New Brunswick's linguistic obligations may be unfulfilled by simultaneous interpretation, rather requiring a French-language interlocutor to provide equal services to both linguistic communities, in the spirit of section 16.1 of the *Canadian Charter of Rights and Freedoms* (Radio-Canada, 2020).

### Québec

Québec shines by comparison in the domain of COVID-19 minority-language communications. Despite *la Charte de la langue française* providing no obligation to do so, daily briefings by Premier François Legault have consistently been held in French and English, without the use of interpreters. However, there have been some gaps in written information distributed to citizens. A “COVID guide” sent to all Québec households was originally available in French only (Chambers, 2020). It was translated into English and distributed in paper form several weeks later (Montreal Gazette, 2020). The *Secrétariat du Québec aux relations canadiennes* (SQRC) has published a special edition of its French-language “COVID guide” for Francophones outside of Québec (SQRC, 2020), filling the gap left by other provinces with respect to minority-language services and demonstrating a form of “kin-state” action on behalf of Québec.

### Other Provinces

Good practices have been witnessed in other provinces. Prince Edward Island's Chief Public Health Officer answered questions in French during briefings, as did British Columbia's Minister of Health (and Minister Responsible for Francophone Affairs). Jason Kenney, premier of Alberta, has also answered questions in French during briefings and gave interviews in that language to various media outlets. Despite less robust language obligations than Ontario and New Brunswick, these provinces have offered information to their Francophone population during these briefings.

## Why Should Language Rights Be Upheld during a Pandemic?

We have identified two main arguments to uphold language rights during the outbreak: public safety and public health.

### Public Safety

Citizens need to have access to information, legislation and regulations coming from numerous stakeholders, like chief medical officers, premiers and ministers. As previously stated, some governments have relied on simultaneous interpretation or closed captioning of briefings relayed on electronic platforms. Yet these are not accessible to all citizens, notably in rural or remote areas. Some argue that since the rate of bilingualism is higher among Francophones in Canada (Statistics Canada, 2017), everyone should understand the guidelines circulated in English. However, French unilingualism is common, especially among the elderly, and English–French bilingualism is often self-evaluated. Francophones tend to overstate their capacity in English in surveys or during interactions with state institutions (Deveau et al., 2009; Tardif and Dallaire, 2010). Therefore, in order to ensure proper understanding of daily directives, access to information in French is paramount. There is anecdotal evidence that French speakers outside of Quebec watch the Quebec briefings to access information in French (Liberal Party of Ontario, 2020). This may prompt citizens to comply with Quebec's guidelines, rather than those of their own province, which could have public safety (and legal) implications.

### Public Health

Language obligations are also important for health reasons. Language barriers when receiving health services can have adverse effects on a patient (Bowen, 2015), such as errors in diagnosis and inadequate subsequent treatment. They can also lead to health promotion and education resources being underused. These risks are higher for vulnerable members of society, among whom are the elderly (OFLSC, 2016). As we know, COVID-19 disproportionately affects seniors, who make up a comparatively larger proportion of the Francophone population in minority communities compared to the majority and who have a low level of literacy, making it harder for them to navigate the health system (Bouchard and Desmeules, 2017). Also, second-language proficiency decreases with age, and stressful conditions and cognitive issues such as dementia are aggravating factors (OFLSC, 2018). It is thus imperative that this segment of the population receive information on COVID-19 in its mother tongue. Some of these observations are also valid for recent Francophone immigrants outside of Québec.

While the issue of non-official languages falls outside the scope of this analysis, we acknowledge that several of the above arguments also apply to government services, and especially communications, in other non-official languages. This would ensure that a larger proportion of citizens is properly informed, including Indigenous language speakers and allophone immigrants.

## Conclusion

Since the beginning of the COVID-19 outbreak, most governments have enacted and communicated policies through daily briefings. Appropriate measures must be implemented to ensure speakers of a minority language are adequately informed. Beyond the state's responsibility to uphold the rule of law and to maintain the public trust in its institutions, the policy basis of public safety and public health outlined above more than justify minority language services throughout an emergency. Canada is not alone in this situation; other minority language communities have observed a similar challenge (Collective, 2020). Governments have already shown they understand the impetus of being understood by all, including the most vulnerable, by engaging sign language interpreters for their briefings.

As Béland et al. (2020) have previously highlighted, COVID-19 has created a critical juncture in the federal state's interactions with the population. Institutions generally resistant to the use of new technologies, for example in the administration of justice or in education, have been compelled to adjust to ensure the delivery of essential services. While institutions are innovating rapidly, language rights and obligations should be an integral part of this transformation, rather than an afterthought or a hindrance. Existing minority-language service delivery bodies should be mobilized by governments in this transition to help plan for consistent inclusion of language rights in government action, now and in the future.
